# Immunological research landscapes and emerging immune mechanisms in HIV/HBV co-infection: a bibliometric analysis (2014–2024)

**DOI:** 10.3389/fimmu.2026.1807430

**Published:** 2026-05-14

**Authors:** Ai Peng, Khalid Waleed, Liping Qiu, Li Wang, Xiaoqin Tong, Zhibin Tu, Zhikang Li, Yiting Cui, Fei Hu, Shu Yang, Liang Lu, Peng Huang

**Affiliations:** 1 Center for Evidence-Based Medicine, Jiangxi Provincial Key Laboratory of Disease Prevention and Public Health, School of Public Health, Jiangxi Medical College, Nanchang University, Nanchang, Jiangxi, China; 2 The Collaboration Unit for State Key Laboratory of Infectious Disease Prevention and Control, Jiangxi Provincial Health Commission Key Laboratory of Pathogenic Diagnosis and Genomics of Emerging Infectious Diseases, Nanchang Center for Disease Control and Prevention, Nanchang, Jiangxi, China; 3 Department of Orthopaedic Surgery, The Second Affiliated Hospital, Jiangxi Medical College, Nanchang University, Nanchang, Jiangxi, China; 4 Department of Cancer Medical Center, The First Affiliated Hospital, Jiangxi Medical College, Nanchang University, Nanchang, Jiangxi, China

**Keywords:** bibliometric analysis, CD4+ T cell dysfunction, chronic immune activation, HIV/HBV co-infection, Immune dysregulation, immune-mediated liver injury

## Abstract

**Background:**

HIV and hepatitis B virus (HBV) co-infection remains a major global health challenge and is characterized by profound immune dysregulation, including chronic immune activation, impaired antiviral immunity, and immune-mediated liver injury. With the widespread use of effective antiretroviral therapy, people living with HIV now experience prolonged survival, shifting clinical and research priorities toward long-term immunological consequences and chronic comorbidities such as HBV. Although a rapidly expanding body of literature has explored HIV/HBV co-infection, a comprehensive immunology-oriented overview of global research trends, collaborative structures, and evolving immune-related research themes is still lacking.

**Methods:**

Publications related to HIV/HBV co-infection published between 2014 and 2024 were retrieved from the Web of Science Core Collection and Scopus databases. After data cleaning and deduplication, 1,649 eligible records were included. Bibliometric analyses were performed using CiteSpace and VOSviewer to evaluate temporal publication trends, international and institutional collaboration networks, journal distributions, keyword co-occurrence patterns, citation bursts, and emerging research frontiers, with a particular focus on immune-related research themes and host–virus immune interactions.

**Results:**

The annual number of publications increased steadily over the study period, with a marked rise in citation activity after 2017, reflecting sustained and growing scientific interest in the immunopathogenesis of HIV/HBV co-infection. High-income countries, particularly the United States and Western Europe, dominated research output and held central positions in global collaboration networks, whereas high-burden regions, such as sub-Saharan Africa, increasingly contributed through international partnerships. Keyword co-occurrence and citation-burst analyses revealed a clear immunological thematic evolution: early studies focused on antiviral therapy and virological suppression, followed by increased attention to epidemiology and clinical guidelines, and more recently a pronounced shift toward immune-related outcomes. Emerging research hotspots increasingly emphasized CD4^+^ T-cell dysfunction, persistent immune activation, immune exhaustion, chronic inflammation, and immune-mediated liver injury, highlighting the growing recognition of immune dysregulation as a central driver of disease progression, fibrosis, and long-term prognosis in HIV/HBV co-infected populations. Further analyses of the journal demonstrated the integration of immunology with virology, hepatology, and infectious disease research.

**Conclusions:**

Global research on HIV/HBV co-infection has evolved from predominantly treatment- and virology-focused studies toward immunology-driven, outcome-oriented, and translational research addressing long-term immune dysfunction and prognosis. Despite expanding international collaboration, substantial disparities persist between regions with high disease burden and those leading immunological research. These findings underscore the need for strengthened locally led, immunology-focused research, long-term cohort studies, and integrated immune-monitoring strategies to improve clinical outcomes for people living with HIV/HBV co-infection.

## 1 Introduction

Co-infection with human immunodeficiency virus (HIV) and hepatitis B virus (HBV) is a serious challenge in the global public health field. Since both viruses are transmitted through blood, sexual contact, and mother-to-child transmission, the infection rate of HBV in HIV-infected patients is significantly higher than that in the general population (1). Studies (2, 3) have shown that HIV/HBV co-infection can accelerate the development of HBV-related liver disease, making patients more likely to progress to liver fibrosis and cirrhosis more rapidly than those infected with HBV alone, and increasing the risk of hepatocellular carcinoma (HCC), with a poor overall prognosis. HBV co-infection may also affect the disease progression of HIV, with complex implications for the choice of antiretroviral therapy (ART) regimen (4).

Over the past decade, research on HIV/HBV co-infection has advanced significantly, particularly in understanding epidemiology, pathogenesis, and clinical management in high-burden settings such as sub-Saharan Africa (5, 6). Recent research has examined viral mutations, HBV genotypic diversity, and the long-term effects of tenofovir-based treatments (7–9). A crucial gap persists despite this scientific advancement: the global distribution of research activity does not appear to align with the burden of disease. Low- and middle-income countries—where HIV/HBV co-infection prevalence is highest—continue to face significant barriers in accessing screening, treatment, and sustained care. Whether international research adequately centers the needs and contexts of these regions remains unclear.

In general, narrative reviews summarize clinical findings. Still, they struggle to convey the larger framework of international research activity, such as regional patterns of research leadership, collaboration networks, or thematic focus areas. A complementary, large-scale method that enables a more objective examination of these research dynamics is bibliometric analysis (10).in this study our goals were to: (1) describe patterns of international collaboration between nations, institutions, and authors; (2) identify key research themes and their evolution over time; and (3) assess whether current scientific output is consistent with the distribution of HIV/HBV disease burden worldwide.

In this regard, the reinforcement of evidence-based HIV/HBV co-infection research is closely interconnected with the United Nations Sustainable Development Goal 3, which aims to ensure healthy lives and well-being for all, and the global plans of the World Health Organization to eradicate HIV and viral hepatitis as a threat to the worldwide population by 2030 (11). To achieve these goals, however, it is necessary to gain a better insight into the immunological processes underlying HIV/HBV co-infection. Immune dysregulation, including CD4+ T-cell depletion, persistent immune activation, and deficiency of HBV-specific immune responses mediated by HIV, is central to sustained viral replication, immune-mediated liver damage, and rapid disease progression (6, 12). Mapping patterns and gaps in global research and knowledge from an immunological perspective may thus serve to define priorities for immune-centric investigation and translation. This understanding is critical to advising the incorporation of immune-based screening, monitoring, and therapeutic strategies into HIV care frameworks, especially in circumstances with high-burden and resource-limited environments wherein immune pathophysiology plays a leading role in unfavorable long-term outcomes (9, 11).

## 2 Methods

### 2.1 Data collection and selection criteria

The bibliometric analysis was performed to examine research trends on HIV/HBV co-infection worldwide. The Web of Science Core Collection (WoSCC) and Scopus are authoritative databases widely used in bibliometric and knowledge-structure visualization studies, as their indexing systems are standardized and provide comprehensive citation data (10, 13). Literature data were found in these two databases (14). Although PubMed is widely used for biomedical literature retrieval, it does not provide the complete citation metadata required for bibliometric mapping and network visualization. The Web of Science Core Collection and Scopus were thus chosen as the primary data sources, as they provide standard citation indexing and comprehensive reference information and are widely used in bibliometric and health research circles.

A topic-based search strategy was used, including titles, abstracts, author keywords, and Keywords Plus. The search date range was limited to January 1, 2014, through December 31, 2024, and all searches and data exports were performed on November 6, 2025, to reduce potential bias from ongoing database updates (13, 14).The time frame of 2014–2024 was selected to capture the most recent decade of HIV/HBV co-infection research, reflecting the period following the widespread implementation of modern antiretroviral therapy (ART). This period is characterized by a shift from virology-focused studies toward long-term clinical outcomes and immunological mechanisms, which aligns with the objectives of the present study. Restricting the analysis to this timeframe also improves data consistency and comparability in bibliometric analysis. The search question to use was HIV- and HBV-related terms, using the Boolean operators in the following way.: (“HIV” OR “human immunodeficiency virus” OR “AIDS” OR “acquired immunodeficiency syndrome”) AND (“HBV” OR “hepatitis B virus” OR “hepatitis B”) AND (“coinfect*” OR “co-infect*” OR “co-infection” OR “dual infect*”), confirming comprehensive retrieval of publications addressing HIV/HBV co-infection.

### 2.2 Inclusion and exclusion criteria

Articles or review articles were included, published in English, targeted on the topic of HIV/HBV co-infection, and published between 2014 and 2024. Publications were excluded if they were editorials, letters, meeting abstracts, book chapters, or proceedings papers. Records that had not been completed with bibliographic details were also left out. Moreover, duplications, invalid records, and articles outside the timeframe were eliminated to ensure data consistency and high methodological rigor. All the qualified records were exported as entire records and reference materials. WoS records were sent in plain-text format, and Scopus records were sent in RIS/CSV format, after which they were converted to Web of Science format via CiteSpace to ensure consistency of bibliographic fields. To ensure the relevance of the dataset and minimize the inclusion of unrelated studies, a topic-based screening process was applied. Retrieved records were evaluated based on titles, abstracts, and keywords to confirm their direct relevance to HIV/HBV co-infection. Publications that did not specifically address HIV/HBV co-infection were excluded during data preprocessing. This multi-step filtering process ensured that the final dataset was accurate and representative, with minimal inclusion of irrelevant noise.

### 2.3 Data preprocessing and dataset construction

**Figure 1:**
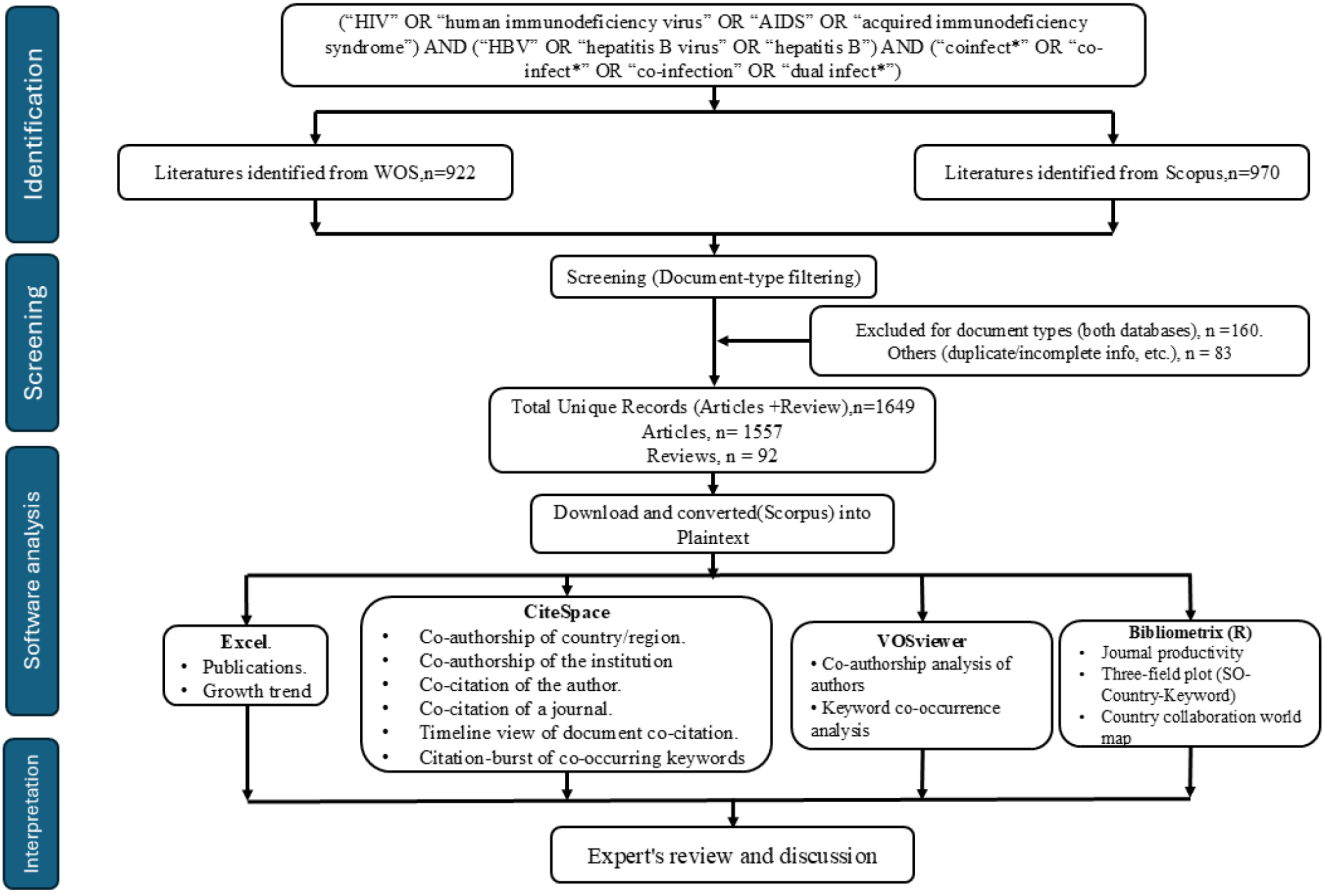
The PRISMA flowchart illustrates the search strategy and selection process in HIV/HBV co-infection.

Both databases were combined into a single folder containing all WoS-format files. Duplicate records were removed based on DOI and title matching using CiteSpace version 6.4.R1 (64-bit), a standard method for integrating databases in bibliometric studies (15). A total of 1892 records were extracted. After removing duplicates and invalid records, only 1,732 unique records remain, of which 920 were produced by Scopus and 812 by WoS, demonstrating that both databases can be successfully integrated as shown in **Figure 1**. To ensure consistent methodology and academic rigor, only articles and review articles were retained in the final analysis. Following the selection of document types and the deletion of hybrid records (early-access and proceedings-related records), the final dataset comprised 1,557 articles and 92 reviews, totaling 1,649 publications.

### 2.4 Bibliometric analysis and visualization

CiteSpace version 6.4.R1 (64-bit) was used to perform knowledge mapping and visualization analysis, a standard bibliographic visualization tool built on scientific knowledge graph theory. VOSviewer was applied to visualize keyword co-occurrence and author collaboration networks. A dual-map overlay was constructed to explore citation relationships between disciplines. Microsoft Excel 2024 was used to conduct descriptive statistical analyses, including the number of journals published and distributed annually, as well as the countries of distribution. In addition, Bibliometrix (R package) and its web-based interface Biblioshiny were used to complement the bibliometric analysis by providing descriptive source-level and temporal analyses.

The time slice was established from 2014 to 2024, with a one-year slice length. Title, Abstract, Author, Keywords, and Keywords Plus were used as term sources. The G-index criterion was used with a scale factor k = 25. The Pathfinder algorithm was used to perform network pruning to enhance the clarity of network visualization (15). It was analyzed in five key areas, including (1) country/region, (2) author, (3) institution, (4) journal, and (5) keywords. The size of a node in any network is its degree, and betweenness centrality is a crucial metric indicating whether a node serves as a bridge in collaboration and knowledge-transfer networks (13, 15). The log-likelihood ratio (LLR) algorithm was used to assign cluster labels, and the keyword burst was used to detect emerging research frontiers. All analytical procedures followed established bibliometric research standards to ensure reproducibility and methodological transparency.

## 3 Results

### 3.1 Evolution of immunology-focused HIV/HBV research output

**Figure 2:**
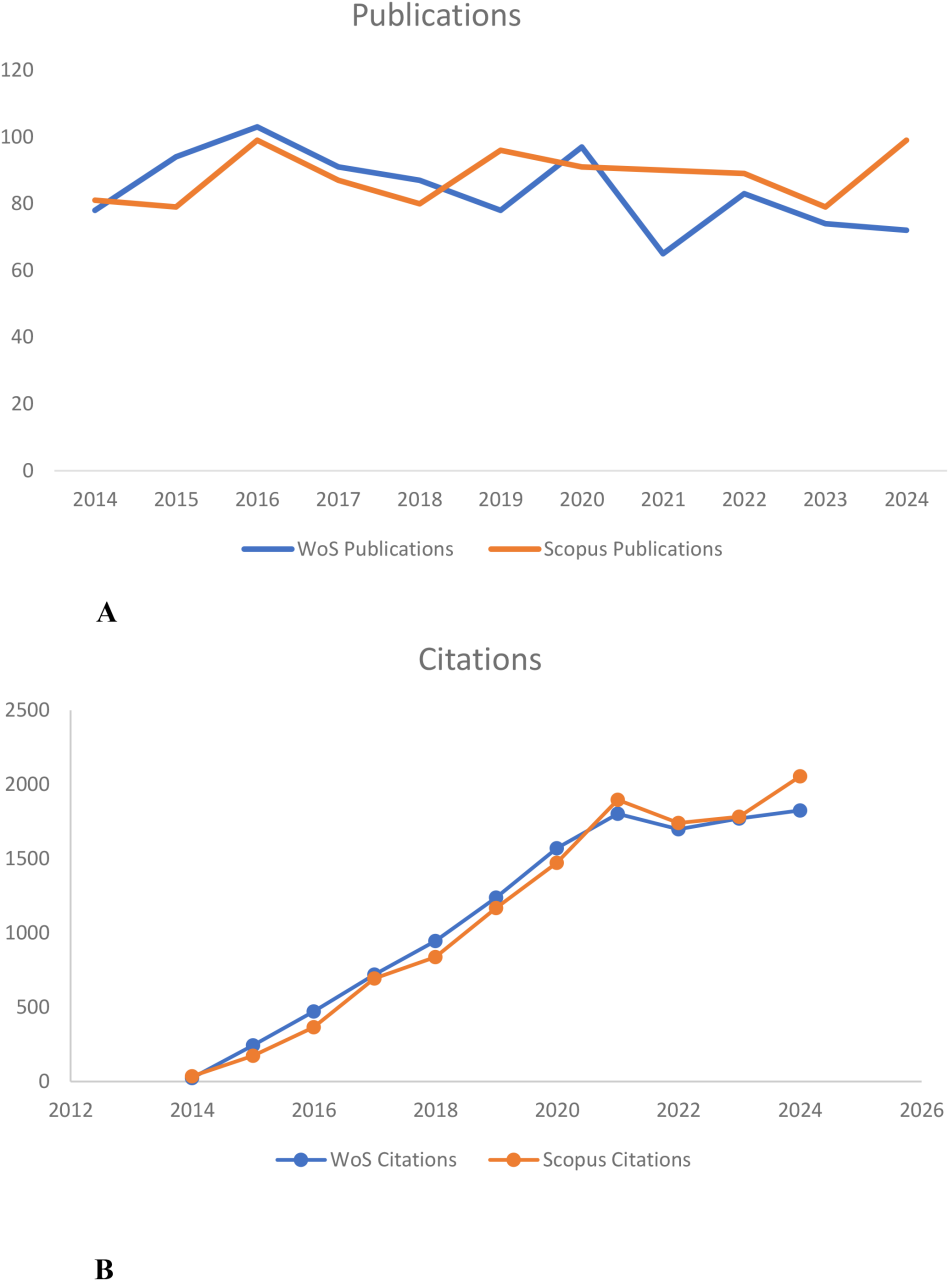
**(A)** Annual publication trends of HIV/HBV co-infection research in WoS and Scopus (2014–2024). **(B)** Annual citation trends of HIV/HBV co-infection research in WoS and Scopus (2014–2024). Note: **(A)** shows yearly publication counts in WoS and Scopus. **(B)** shows corresponding annual citation frequencies. Data were retrieved from the Web of Science Core Collection and Scopus databases.

The trend in the annual number of publications on HIV/HBV co-infection research indicates a stable, sustained increase from 2014 to 2024 (**Figure 2A**). In general, Scopus has always indexed more publications than WoS across all years. The number of journals published per year grew slowly following 2014 and peaked early in 2016 in each database. Even though there was a slight decline after 2017, the number of annual publications remained relatively high, suggesting that this scientific area remains well-studied.

The frequency of citation in both WoS and Scopus, as reported in **Figure 2B**, has been soaring upward since 2017. The number of citations increased rapidly through 2020 and has since risen to approximately 2,055 in Scopus and 1,826 in WoS. Combined with the steady rise in the volume of published articles and a sharp increase in citations, these patterns indicate increasing scientific influence and sustain global research interest.

### 3.2 Global distribution of immunological research leadership

The studies of HIV/HBV co-infection indicate a broad international involvement, as over 100 countries and regions have been involved in this field. Considering merged and deduplicated data (N = 1649), the United States leads in publications (N = 421; 25.53%) and in betweenness centrality (0.35), indicating its dominant role and strong bridging position within the global collaboration network. China ranks second with 189 publications (11.46%), suggesting a rapidly growing research presence but relatively low centrality in the network. France has the third largest number of publications (151) (9.16%), and a high value of centrality (0.29), which reflects its centrality in international cooperation. The United Kingdom ranks fourth (109 publications, 6.61%) and is also a key collaborative center within the global research system.

The other nations also play an essential role in the global research scene. South Africa boasts 104 articles (6.31%), a significant centrality, and is expected to have a high regional burden of HIV/HBV co-infection, and is actively involved in African research networks. Italy (100 publications), Brazil (81 publications), Canada (80 publications), India (78 publications), and Iran (72 publications) further enhance global scientific cooperation and create additional regional research centers. These results demonstrate a research-based world that is both interconnected and uneven, with high-income nations predominant in producing scientific evidence and high-burden regions becoming increasingly interconnected with international partners.

**Figure 3:**
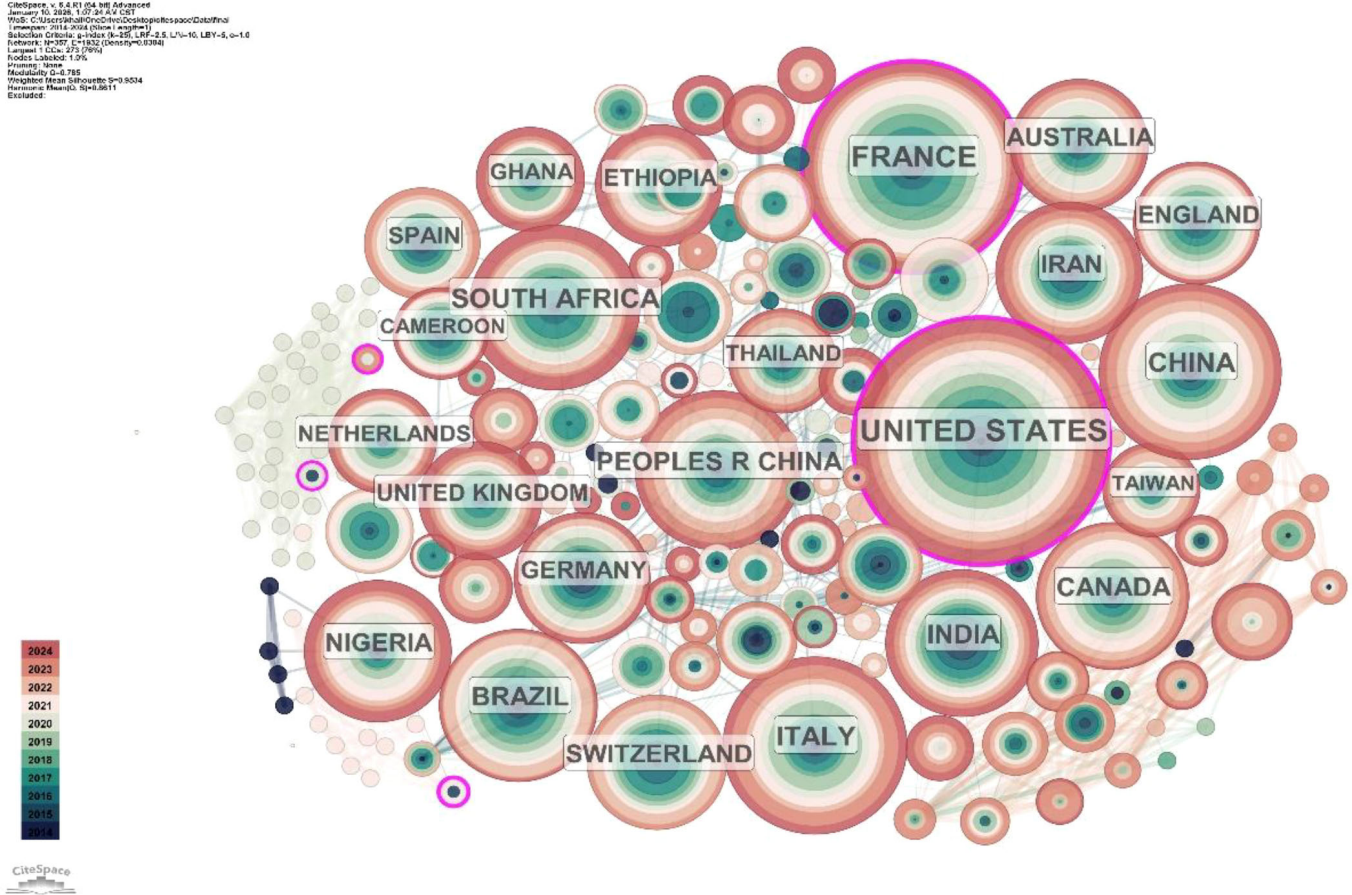
Cooperation map of countries/regions in HIV/HBV co-infection. A visual map for the CiteSpace network. Note: Node size represents publication volume, links represent international collaboration, and purple rings indicate high betweenness centrality. Color gradients reflect the progression of publication time from 2014 (blue) to 2024 (red).

The national cooperation network generated by CiteSpace is shown in **Figure 3**. The size of the node is proportional to the number of publications, with the United States having the largest node. The purple rings denote high betweenness and centrality, highlighting countries that serve as essential bridges in multinational cooperation. The degree of collaboration among states is seen in the thickness of links. The network demonstrates strong interconnections among North America, Western Europe, East Asia, Africa, and South America, depicting a highly organized, more complex network of researchers worldwide.

**Figure 3** is quantitatively supported by **Table 1**, which summarizes the number of publications and centrality values for the most prolific countries/regions, providing a clear picture of research outputs and the structural significance of these countries/regions in the international collaboration network.

**Table 1:** Top 10 productive countries/regions in HIV/HBV co-infection.

Rank	Country/region	Publications	(%) of 1649	Centrality
1	United States*	421	25.53	0.35
2	China*	189	11.46	0.02
3	France	151	9.16	0.29
4	United Kingdom*	109	6.61	0.02
5	South Africa	104	6.31	0.05
6	Italy	100	6.06	0.00
7	Brazil	81	4.91	0.00
8	Canada	80	4.85	0.01
9	India	78	4.73	0.01
10	Iran	72	4.37	0.00

*Country names appearing under multiple database labels were unified for publication counts. (e.g., “USA” + “United States”; “China” + “Peoples R China”; “UK” + “England”).Centrality values are reported directly from CiteSpace for the dominant corresponding node label, as betweenness centrality cannot be arithmetically merged without recalculating network topology.

### 3.3 Core immunology research networks

**Figure 4:**
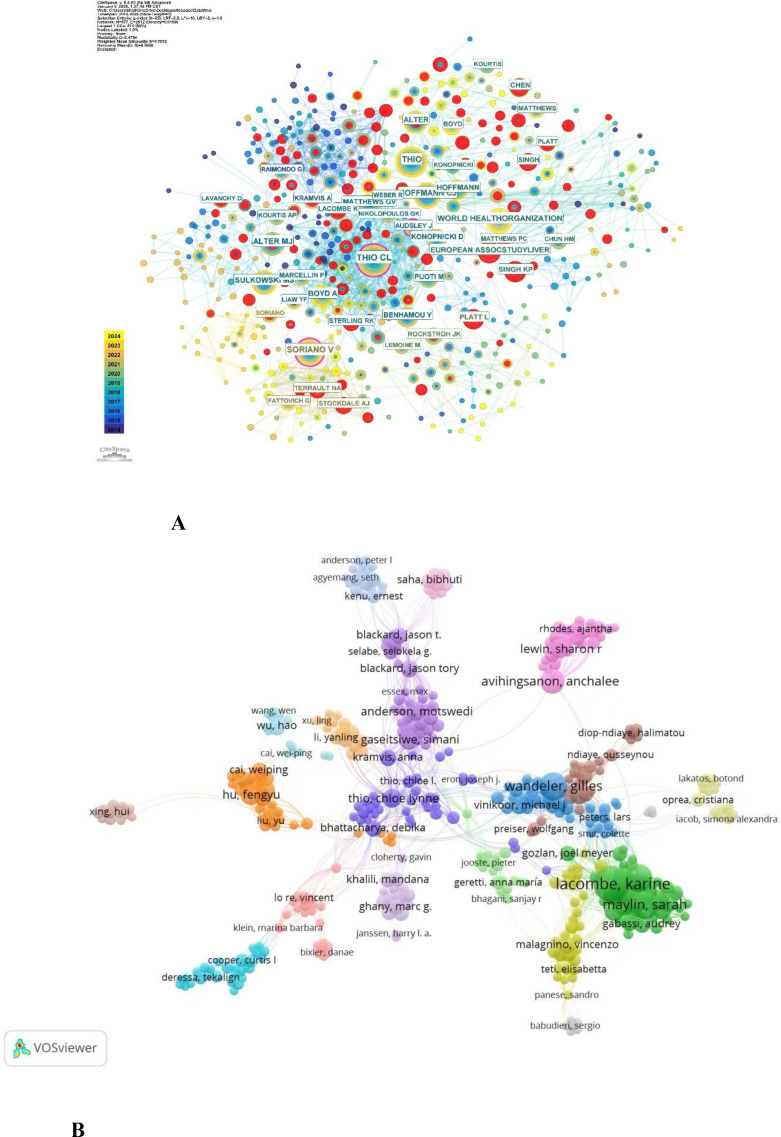
**(A)** Author collaboration network by Citespace. Note: Node size represents publication volume; links indicate co-authorship relationships. Purple rings denote high betweenness centrality. **(B)** Author collaboration network by VOSviewer. Node size represents publication output, and links indicate collaborative relationships between authors.

**Figure 5:**
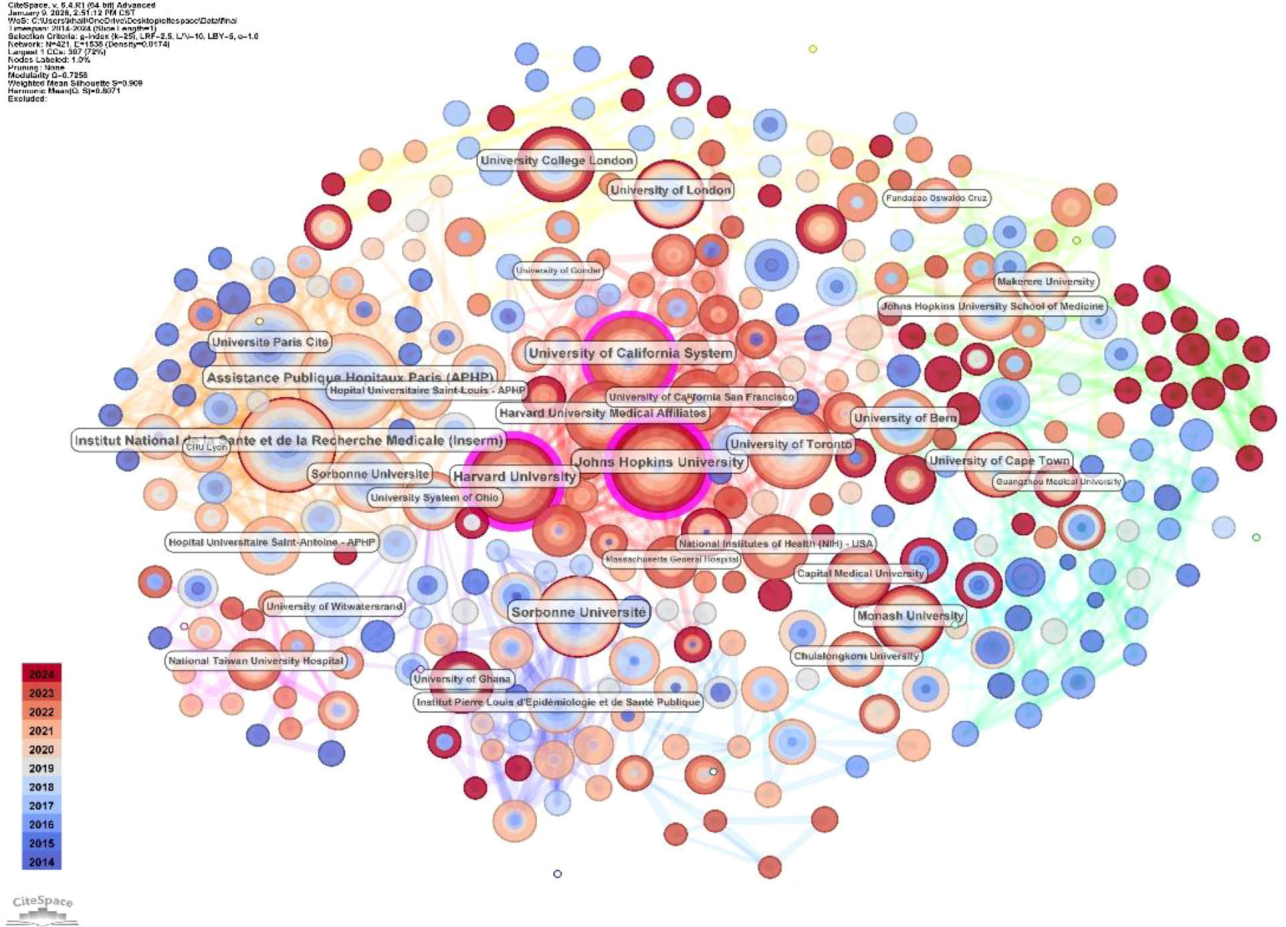
Immunology-relevant journals and knowledge flow. Note: Node size represents institutional publication output; links indicate inter-institutional collaboration. Purple rings denote high betweenness centrality.

The publication frequency and network structural indicators derived from the combined WoS-Scopus dataset were used to systematically examine research productivity and collaboration patterns at the author and institutional levels in HIV/HBV co-infection research (**Table 2**, **Figure 4**-5). According to **Table 2A**, Thio CL had the highest number (132), accounting for 8.00% of the total literature; Soriano V (96, 5.82) and Hoffmann CJ (88, 5.34) followed. The World Health Organization also played a significant role, with 81 publications (4.91%), and coordinates global work on HIV/HBV. Alter MJ (74 publications), Sulkowski MS (63 publications), and Boyd A (62 publications) are other highly productive authors, each with a relatively narrow core group of researchers who exert significant influence in this area. The betweenness centrality values are also relatively high for these authors, implying an essential role in linking various collaborative sub-networks and facilitating knowledge exchange.

**Table 2:** Top 10 authors and research institutions in HIV/HBV co-infection research.

A. Top 10 authors				
Rank	Author	Publications	(%) of 1649	Centrality
1	Thio CL	132	8.00	0.12
2	Soriano V	96	5.82	0.13
3	Hoffmann CJ	88	5.34	0.07
4	World Health Organization	81	4.91	0.04
5	Alter MJ	74	4.49	0.05
6	Sulkowski MS	63	3.82	0.08
7	Boyd A	62	3.76	0.08
8	Audsley J	39	2.36	0.17
9	Kramvis A	36	2.18	0.14
10	Matthews GV	35	2.12	0.11
B. Top 10 Research Institutions				
Rank	Institution	Publications	(%) of 1649	Centrality
1	Assistance publique–Hôpitaux de Paris (AP-HP)	43	2.61	0.07
x2	INSERM (Institut national de la santé et de la recherche médicale)	42	2.55	0.04
3	Johns Hopkins University	41	2.49	0.20
4	Harvard University	36	2.18	0.24
5	University of California System	34	2.06	0.08
6	University of Toronto	34	2.06	0.13
7	Université Paris Cité	33	2.00	0.01
8	University College London	31	1.88	0.07
9	University of London	29	1.76	0.10
10	Sorbonne Université	28	1.70	0.04

**(A)** lists the top 10 most productive authors, ranked by publication count and betweenness centrality. **(B)** lists the top 10 most productive research institutions. Percentages indicate the proportion of total publications (n = 1649). Betweenness centrality reflects the bridging role of authors or institutions within collaboration networks.

The collaboration network generated for the authors in CiteSpace is presented in **Figure 4A**. Some authors with medium publication rates yet high centrality values are bridging nodes between various research clusters, which contributes to the structural cohesion of the global collaboration network. At the institutional level (**Table 2B**), Assistance Publique-Hôpitaux de Paris (APHP) led in publications, with 43, followed by INSERM (42) and Johns Hopkins University (41). The University of California System (34 publications) and Harvard University (36 publications) also showed high productivity and centrality, indicating that researchers play a central role in global collaborative research. University College London, Université Paris Cité, and the University of Toronto, among other institutions, also contributed to the formation of a multi-centered international research structure.

Author collaboration network analysis identified several distinct research communities with clear cluster structures (**Figure 4B**). Several highly productive authors occupied central positions in the network, indicating established research hubs in HIV/HBV co-infection research. Collaboration was strong within clusters, while inter-cluster connections were relatively limited.

**Figure 5** depicts the institutional collaboration network, with node size reflecting publication volume and purple rings indicating high betweenness centrality. It is found that a highly interconnected core group links major institutions in North America and Europe, and that these connections are expanding to institutions in Asia and Oceania. This indicates the existence of a research space that is distributed globally yet structurally unified. These data suggest that HIV/HBV co-infection studies are conducted by a relatively stable group of most prolific writers and institutions that large global networks of collaboration can reinforce.

### 3.4 Journal distribution

HIV/HBV co-infection research is concentrated in a limited number of high-output journals (**Table 3**). Most leading journals are classified in Q1 or Q2, with impact factors ranging from approximately 2.5 to 6.0, indicating substantial academic influence and visibility in this research field. PLOS ONE ranked first in publication output with 125 articles (7.58%), followed by BMC Infectious Diseases (74 articles; 4.49%) and the Journal of Viral Hepatitis (45 articles; 2.73%). Together, these three journals accounted for 14.80% of all publications, making them the primary venues for research on HIV/HBV coinfection. Other journals with consistent contributions included the Journal of Medical Virology, AIDS, the International Journal of Infectious Diseases, and Liver International, reflecting the interdisciplinary nature of this research area across infectious diseases, virology, and hepatology.

**Table 3:** Top 10 journals in HIV/HBV co-infection.

Rank	Journal	Publications	(%) of 1649	Centrality	IF (2024)	JCR (2024)
1	PLOS ONE	125	7.58	0.00	2.6	Q2
2	BMC Infectious Diseases	74	4.49	0.01	3.0	Q2
3	Journal of Viral Hepatitis	45	2.73	0.00	4.6	Q3
4	Journal of Medical Virology	38	2.30	0.01	4.6	Q1
5	AIDS	25	1.52	0.00	3.1	Q2
6	International Journal of Infectious Diseases	24	1.46	0.01	4.3	Q1
7	Liver International	24	1.46	0.01	5.2	Q1
8	Journal of Clinical Virology	23	1.39	0.01	3.4	Q2
9	African Health Sciences	23	1.39	0.03	0.89	Q3
10	BMC Public Health	21	1.27	0.01	3.6	Q1

lists the top 10 source journals ranked by publication counts in the combined WoS–Scopus dataset (n = 1649). IF indicates the 2024 Journal Impact Factor, and JCR represents the Journal Citation Reports quartile classification.

**Figure 6:**
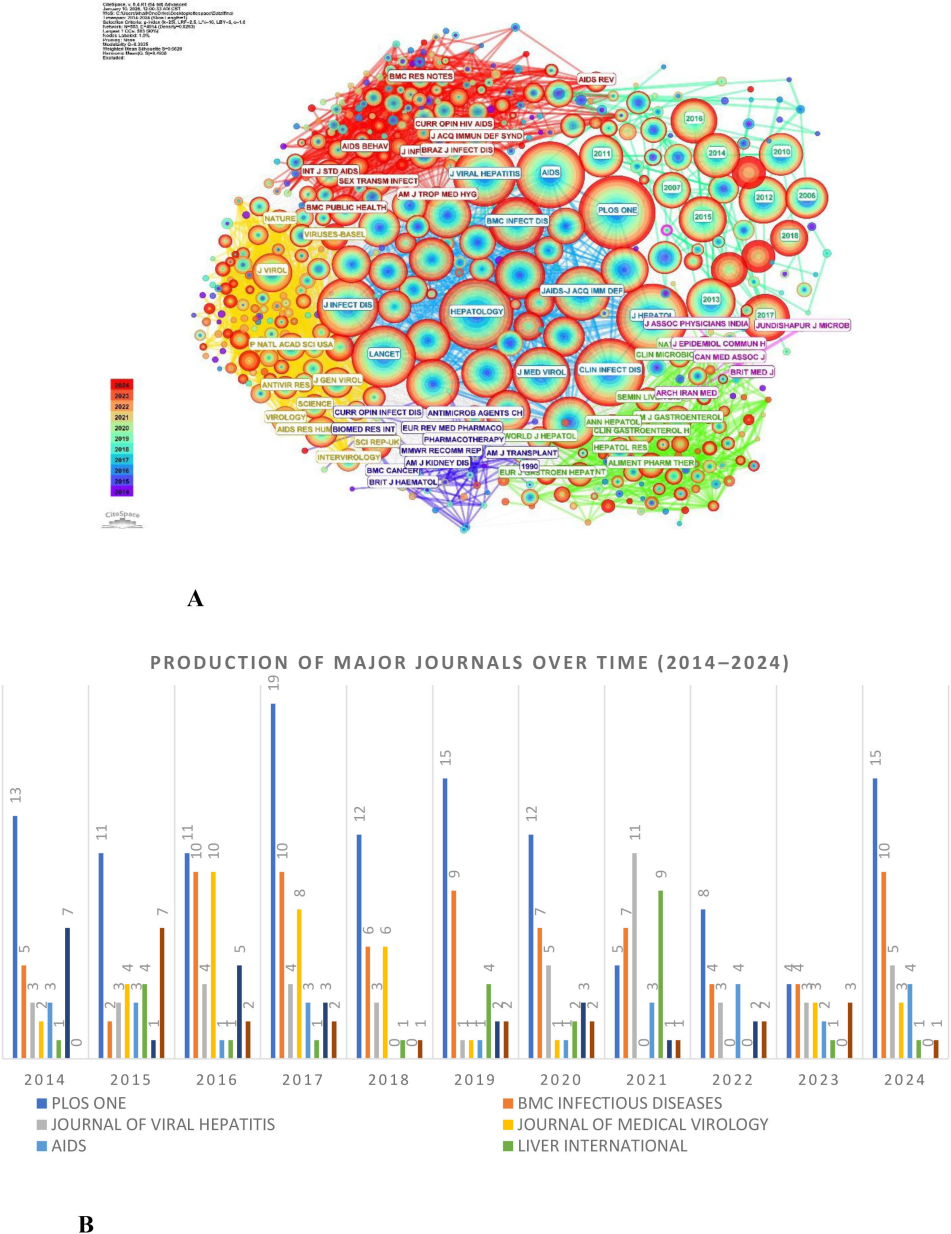
**(A)** Journal distribution of HIV/HBV co-infection. Note: Nodes represent journals; node size is proportional to publication volume; link thickness indicates co-citation frequency; purple rings denote high betweenness centrality; node colors represent publication year evolution (2014–2024). **(B)** Annual publication output of leading journals in HIV/HBV co-infection research (2014–2024).

**Figure 6A** presents the journal co-citation network of HIV/HBV co-infection studies. In this network, nodes represent journals, with node size proportional to co-citation frequency. At the same time, purple outer rings indicate higher betweenness centrality, highlighting journals that serve as bridges across different research themes. The dense interconnections among journals in clinical medicine, virology, infectious diseases, and public health demonstrate a highly integrated citation structure, confirming the multidisciplinary foundation of HIV/HBV co-infection research.

**Figure 6B**, generated using Bibliometrix, illustrates the publication output of the most productive journals over time. This figure complements the co-citation analysis by depicting the temporal distribution and sustained contributions of core journals throughout the study period, further emphasizing the dominance and continuity of leading publication sources in this field.

Additional visualizations illustrating journal dominance and the structural relationships among source journals, contributing countries, and high-frequency keywords are provided in the Supplementary Materials (Supplementary **Figure 1**–S2).

### 3.5 Immune-related keyword clusters and mechanistic themes

High-frequency keywords were filtered out of the article titles, abstracts, and author keywords to determine the key areas of research in HIV/HBV co-infection. General preset terms (e.g., HIV, HBV, co-infection) were excluded to enhance thematic resolution. The other keywords were explored using co-occurrence network mapping, and their frequencies and network properties are summarized in **Table 4**.

**Table 4:** Top 20 keywords in HIV/HBV co-infection.

Rank	Keywords		Total link strength	Occurrences	Rank	Keywords	Total link strength	Occurrences
1	prevalence	34		198	11	co-infection	41	92
2	human	48		184	12	hepatitis c	40	65
3	hepatitis b virus	36		173	13	seroprevalence	33	63
4	antiretroviral therapy	43		138	14	HBV	45	60
5	infection	31		130	15	infected patients	34	57
6	HIV	43		124	16	mortality	43	56
7	hepatitis b	29		117	17	viral hepatitis	44	56
8	epidemiology	42		100	18	therapy	28	52
9	hepatitis c virus	45		97	19	risk factors	40	49
10	risk	35		97	20	impact	46	48

The keyword frequency represents the number of occurrences of each term across titles, abstracts, and author keywords. Total link strength reflects the overall co-occurrence intensity of each keyword within the network. Keyword analysis was performed using CiteSpace.

Keywords such as prevalence, human, hepatitis B virus, antiretroviral therapy, and infection are high frequency, indicating that the available literature centers on disease burden, treatment methods, and clinical outcomes. Sustained interest in population-level patterns and prognosis is also reflected in other commonly used terms, such as epidemiology, risk, mortality, and viral hepatitis.

**Figure 7:**
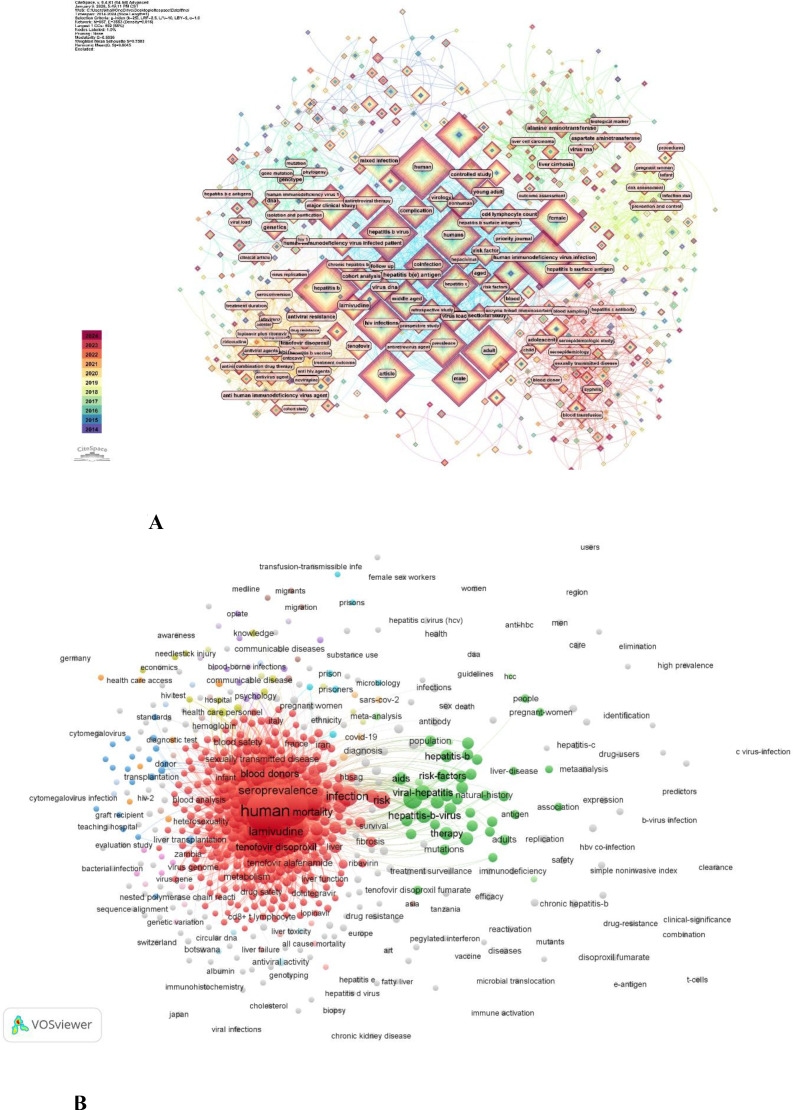
**(A)** The citespace cluster of keywords in the studies of HIV/HBV co-infection. Note: Node size reflects keyword frequency; link thickness indicates co-occurrence strength; node color represents publication time slices from 2014 to 2024. **(B)** The VOS viewer cluster of keywords in the studies of HIV/HBV co-infection. Node size reflects keyword frequency, and links represent co-occurrence relationships.

In addition to frequency, network connectivity analysis revealed several biologically significant immune-based clusters (**Figures 7A, B**). First, keywords such as infection, mortality, and impact characterized an immune activation and chronic inflammation cluster, in which persistent inflammation was identified as contributing to disease development. Second, a cluster of T-cell dysfunction and immune exhaustion terms emerged around HIV-related, patient-related, and antiretroviral therapy-related terms and indicated increased focus on incomplete immune recovery despite viral suppression.

Third, terms such as hepatitis B virus, viral hepatitis, and liver stiffness were clustered and were associated with immune-mediated focal liver injury and fibrosis, underscoring the importance of immune-mediated hepatic damage rather than direct viral injury. Lastly, a cluster of host virus immune interaction, which included HBV, hepatitis C virus, and co-infection, highlighted the multifaceted complexity of the interaction of the virus infection and impaired immune regulation in co-infected persons.

Overall, the analysis of co-occurring keywords indicates a clear shift in research toward immunology-focused topics, with a growing emphasis on immune dysregulation, chronic inflammation, and immune-mediated organ damage in HIV/HBV co-infection.

### 3.6 Emerging immune frontiers and translational immunology hotspots

**Figure 8:**
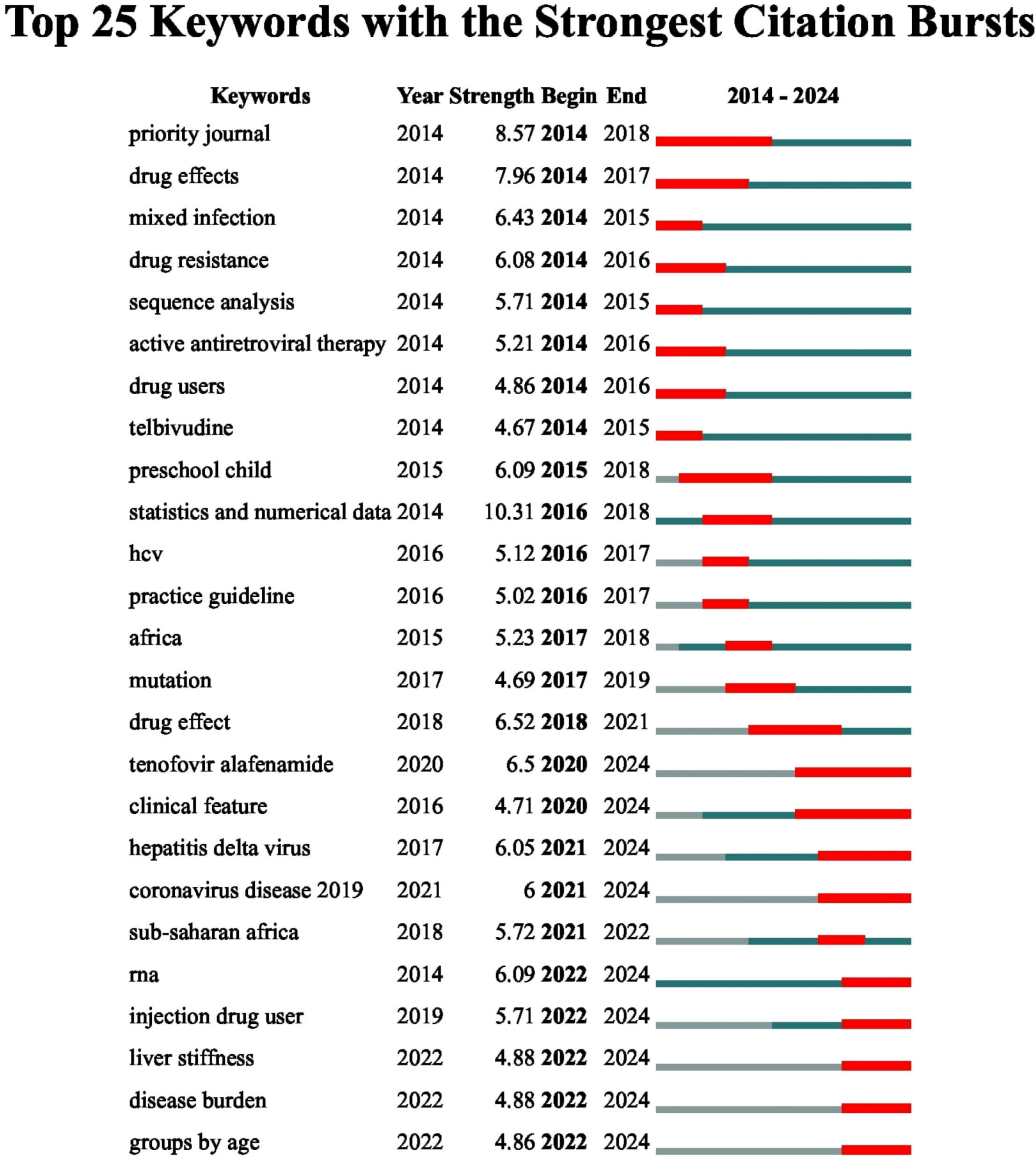
Top 25 keywords with the strongest citation bursts. Note: Red bars indicate time intervals when keywords experienced citation bursts, representing periods of intensified scholarly attention. Burst strength reflects the intensity of research focus. The analysis was conducted using CiteSpace.

CiteSpace was used to identify keywords that experienced significant citation bursts between 2014 and 2024 to understand better the temporal development of new research frontiers in HIV/HBV co-infection. Citation burst analysis identifies terms that have experienced a sudden surge in academic interest within a given period, thereby indicating the emergence of research hotspots. **Figure 8** shows the top 25 burst keywords and the time when they were active. According to **Figure 8**, the first burst period (2014-2016) was characterized by treatment-related and virological mechanism keywords, including priority journals, drug effects, mixed infections, drug resistance, sequence analysis, active antiretroviral therapy, drug users, and telbivudine. These initial bursts represent the initial research focus on the antiviral drug selection, resistance surveillance, and virological features in HIV/HBV co-infection.

The bursts between 2016 and 2019 were oriented toward epidemiological growth and regional interest, with keywords such as practice guideline, Africa, HCV, mutation, and drug effect. This phase suggests increasing interest in developing clinical guidelines, monitoring viral mutations, and assessing the epidemiological impact of co-infection in high-impact regions, notably sub-Saharan Africa. Since 2020, studies have shifted their focus to new therapeutics, clinical characteristics, and disease burden. The keywords, including tenofovir alafenamide, clinical features, hepatitis delta virus, and coronavirus disease 2019, exhibited high and sustained peaks between 2020 and 2024, indicating increased interest in newer antiviral regimens, coinfections with emerging viruses, and the characterization of clinical phenotypes. The more recent burst terms, such as injection drug user, liver stiffness, disease burden, and age (2022-2024), indicate a current direction in population stratification, comorbidity measurement, and long-term prognosis measurement.

In general, the picture of burst keyword development shows evident three-step development of the HIV/HBV co-infection issue research in the last decade: 2014–2016: Early focus on antiviral therapy and viral resistance mechanisms; 2016–2019: Expansion toward epidemiology, guideline development, and regional disease burden; 2020–2024: Current frontiers emphasizing novel antivirals, clinical outcome assessment, comorbidity interactions, and disease burden evaluation. This is a progressive transition, indicating that HIV/HBV co-infection studies have matured and diversified their research to focus on clinical precision and long-term outcomes, and are finally reaching the stage of population-level epidemiological awareness.

### 3.7 Dual-map overlay analysis

**Figure 9:**
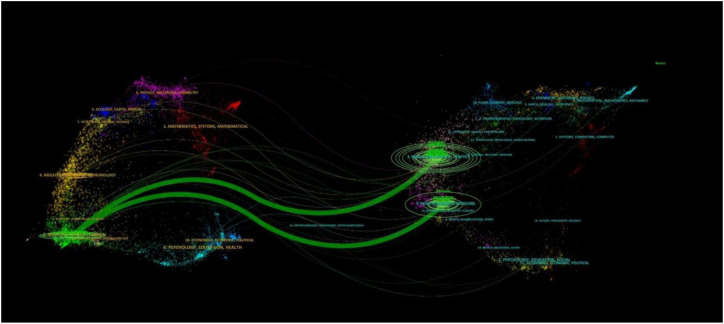
Dual-map overlay of journals in HIV/HBV co-infection research. Left panels represent citing journals, right panels represent cited journals, and curved paths indicate citation relationships.

Dual-map overlay analysis illustrated the citation relationships between journals involved in HIV/HBV co-infection research (**Figure 9**). The primary citation pathways originated from journals in molecular biology and medicine. They were directed toward journals in clinical medicine and public health, indicating a dominant translational flow from biomedical research to applied clinical and population-level studies.

## 4 Discussion

### 4.1 Temporal trends and evolution of HIV/HBV co-infection research

This work is the first bibliometric analysis to conduct a systematic review of research and knowledge development on HIV/HBV co-infection worldwide during 2014-2024. This study combines large-scale data on publications and citation patterns to present a longitudinal perspective on the development of scientific interest in HIV/HBV co-infection over the past 10 years. The characteristics of the included publications, including publication trends, country contributions, and thematic patterns, are presented through bibliometric indicators and visualization analyses.

The findings indicate that the number of published works per year and the number of citations have been gradually increasing since 2017, suggesting that HIV/HBV co-infection research has already become a stable, continuously growing area (12, 16). Since 2017, citation counts have risen steadily, suggesting increasing consolidation of evidence in this field. This continues to expand and is closely related to significant changes in HIV care and global health policies (11, 17). Antiretroviral therapy has become very common and has turned HIV infection into a chronic, treatable condition, which has resulted in better survival and increased life expectancy. This has resulted in chronic comorbidities to gain clinical relevance among HIV-infected individuals, which has prompted sustained research efforts on HIV/HBV co-infection (18, 19).

On the policy level, the international systems and mechanisms are increasingly focusing on comprehensive strategies for the prevention, screening, and long-term management of HIV and viral hepatitis, especially in the context of resource-constrained settings (11, 20, 21). The strategies have established a long-term need for evidence to address co-infection epidemiology, clinical outcomes, and service delivery models, as evidenced by the sustained growth of the research literature. In addition to the quantitative development, the thematic components of HIV/HBV co-infection studies have also changed. Previous research has primarily focused on estimating prevalence and population-level disease burden (22, 23). Conversely, the more recent literature has moved to cover long-term liver outcomes, therapy efficacy, and standard clinical treatment in HIV care initiatives, particularly in high-endemic areas like sub-Saharan Africa (22, 23).

Epidemiological data on HIV clinics in Africa have shown that descriptive surveillance is being gradually replaced by programmatic research, such as HBV testing behaviors, longitudinal follow-ups, and incorporating hepatitis services into the existing HIV services (23–25). This development represents a broader shift in research toward strengthening health systems and long-term patient control. In brief, the temporal trends revealed in this paper may indicate that HIV/HBV co-infection studies have shifted from descriptive epidemiology to clinically and programmatically focused studies. This field maturation provides a critical basis for interpreting the structures of collaboration and the thematic change discussed in the following sections.

### 4.2 Interpretation of key bibliometric findings

The bibliometric findings indicate the presence of a well-organized international study of HIV/HBV co-infection. The most active countries in the production of publications are those with a highly developed research base, particularly the United States and some countries in Western Europe, which hold leading positions in the global network of collaboration. Their centrality betweenness scores indicate that the countries are crucial hubs for knowledge sharing and coordination, influence research directions, and connect various regional research networks rather than merely generating a large number of publications (11, 17).

Conversely, areas with a high burden of HIV/HBV co-infection, most notably sub-Saharan Africa, contribute a smaller percentage of total publications but participate in collaborative research networks that are on the rise. The given pattern is based on the observation that partnerships with institutions in high-income countries drive a significant share of the work undertaken by high-burden settings. Although such collaborations are necessary to produce high-quality data and build local research capacity, they also highlight existing asymmetries in disease burden and research leadership, which have been well documented in global HIV and viral hepatitis research (19, 23, 26).

Notably, European and North American countries are the primary research centers, which may affect the agenda and research priorities. In low-burden contexts where institutions primarily organize collaboration networks, research questions can be more biomedical or methodological than context-specific with respect to implementation barriers to health systems in high-burden settings. This likely mismatch between the global research agenda and local healthcare requirements has been identified as a structural constraint in global health research in general and warrants close attention with respect to bibliometric dominance patterns (23, 27).

The overall control of publications by a relatively small number of researchers and large academic centers, at both the author and institutional levels, is another factor that contributes to the stability of research centers. These centers are major in planning multicenter studies, maintaining long-term cohorts, and providing evidence used to inform clinical guidelines and policy debates (11, 28). Though such concentration can increase the rigor and continuity of methods, it can also constrain the range of views and undercut the visibility of locally motivated research questions in resource-constrained healthcare environments (29).

The patterns of journal distribution will provide further insight into the nature of the field. The fact that the majority of journals devoted to infectious diseases, virology, hepatology, and public health indicate the multidisciplinary nature of research on HIV/HBV coinfection. Simultaneously, the availability of open-access journals is high, and this could indicate a growing focus on disseminating extensive evidence, an essential requirement to meet the needs of policy-making and clinical practice in diverse health care settings. The trend is consistent with global policies of facilitating available evidence to facilitate combined care of HIV and viral hepatitis (11, 30).

### 4.3 Thematic shifts and research progression

**Figure 10:**
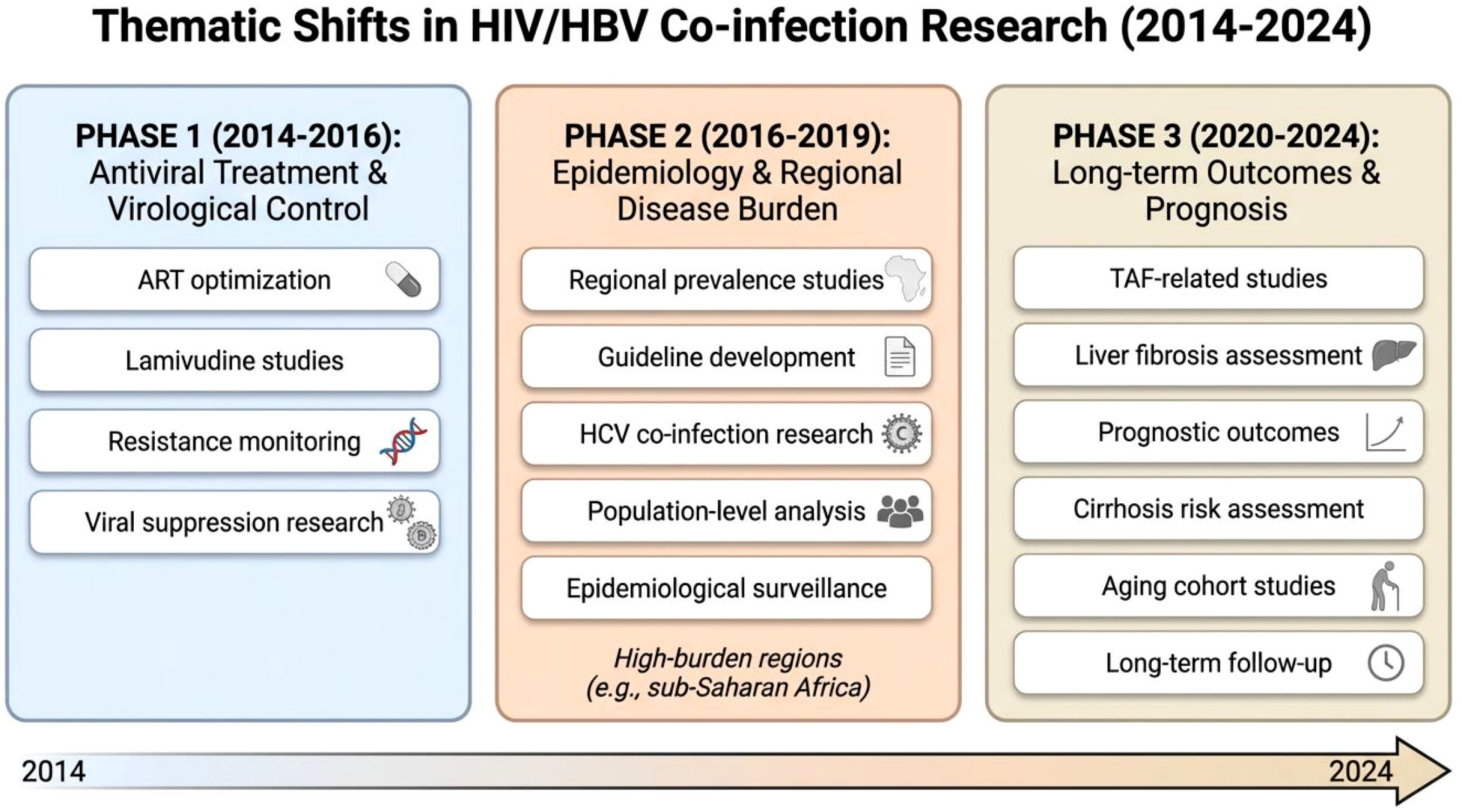
Thematic evolution of HIV/HBV co-infection research (2014–2024).

In recent years, co-occurring keywords and citation bursts analysis show that there has been an evident temporal change in research on HIV/HBV co-infections. Such thematic transformations are related to the changing clinical practice trends and long-term disease management policies (31). The Results indicate three major phases in the field’s evolution, as shown in **Figure 10**, each addressing distinct research questions and clinical environments.

#### 4.3.1 Early phase (approximately 2014–2016): focus on antiviral therapy and virological control

The research focused on the early years, with an emphasis on antiviral treatment approaches and virological processes. High-frequency terms such as ‘antiretroviral therapy,’ ‘lamivudine,’ and ‘drug resistance’ that dominated this period were primarily concerned with research. During this period, research focused on optimizing antiviral regimens to achieve sustained suppression of both HIV and HBV, with the resistance observed against previously used antiviral agents. Clinicians were then mainly worried about the lamivudine resistance and incomplete HBV suppression in co-infected people (18, 28).

Studies published in clinical literature during this period focused on treatment effectiveness, resistance, and virology, particularly in settings with limited access to tenofovir-based regimens. The presence of occult HBV infection in HIV patients who are already receiving therapy only complicates the initial treatment paradigms, which is why monitoring viral mutations is of great significance (32). They are reported in the initial cohort and clinical reviews, underscoring the need for improved antiviral measures to achieve prolonged inhibition of both viruses (6, 11, 18).

#### 4.3.2 Intermediate phase (approximately 2016–2019): expansion toward epidemiology and guideline-oriented research

The middle period became less treatment-based and more focused on epidemiology, disease burden in the area, and guideline development. The fact that there are bursts of citations of the following words/terms: African continent, practice guideline, mutation, and hepatitis C virus, evidence that there is an increased concern about population-level tendencies and the presence of regional peculiarities regarding the treatment of HIV/HBV co-infection. This transformation has been coupled with the tremendous expansion of the antiretroviral therapy interventions in the high-burden locations and increasing recognition of the HBV screening and surveillance gaps in the routine HIV care (24, 34).

Other studies carried out in Sub-Saharan Africa during this period have progressively examined the coverage of HBV tests, viral interaction, and liver outcome in people who are under long-term care treatment of HIV. These studies showed that clinical practice was highly varied and that there was a necessity to have a common set of screening and management interventions in HIV programs (23, 24, 33). This led to a shift in the research focus from antiviral efficacy to health system performance and service delivery.

#### 4.3.3 Recent phase (approximately 2020–2024): emphasis on long-term outcomes and prognosis

During the most recent step, the research priorities were once again revised to place greater emphasis on long-term outcomes and prognostic assessment. The list of included terms includes tenofovir alafenamide, liver stiffness, disease burden, age, and clinical features, indicating increased interest in the development of chronic liver disease, fibrosis assessment, and patient stratification. This trend reflects the clinical reality that increased survival with HIV has shown the morbidity and mortality of HBV co- infection into older age groups, primarily liver-related (9, 25, 28).

Recent cohort and clinical trials have revealed that despite the long-term antiviral therapy, which has improved viral control and lowered the rates of liver-related complications, a residual risk, such as fibrosis and hepatocellular carcinoma, remains (17, 34). The findings have caused growing interest in the non-invasive assessment of fibrosis, the chronic follow-up, and risk stratification models tailored to aging HIV/HBV co-infected individuals.

### 4.4 Clinical and translational implications of immune dysfunction in HIV/HBV co-infection

The identified thematic development in the current bibliometric analysis has significant clinical and translational implications, particularly when viewed from an immunological perspective. These trends highlight the central role of persistent immune activation, CD4^+^ T-cell dysfunction, and immune exhaustion in driving liver pathology and disease progression in HIV/HBV co-infection (34–36). Even under long-term antiretroviral therapy and effective viral suppression, dysregulated host immune responses continue to promote chronic inflammation and immune-mediated hepatic injury, underscoring the limitations of virology-centered management alone. These findings indicate that clinical care should extend beyond virological outcomes to include long-term immunological outcomes and liver prognosis into routine management of HIV (37, 38).

In a clinical view, the persistence of keywords of treatment, including antiretroviral therapy and tenofovir-based regimens, highlights the sustained relevance of dual-active antiviral treatment in the management of both HIV and HBV. The simultaneous introduction of outcome-related terminology, such as liver stiffness, age, and disease burden, indicates that viral suppression alone is insufficient to eradicate the chronic hepatic risk. The mounting clinical evidence suggests that the progression of immune-mediated fibrosis and liver morbidity can still be sustained despite the sustained use of antiviral therapy, especially in patients who become aged with the continued immune perturbation (25, 28).

These results demonstrate the value of chronic inflammation and immune exhaustion in promoting liver pathology and justify continued immunologically wise surveillance. Within translational research, the growing prominence of immune-related clinical phenotypes and patient stratification indicates a greater emphasis on precision-based management. Immune biomarkers, noninvasive markers of immune-mediated fibrosis, and longitudinal immune monitoring should be combined to identify individuals at increased risk of adverse liver outcomes. The introduction of non-invasive methods may facilitate fibrosis evaluation, and long-term follow-up planning can help identify progressive liver disease earlier and provide a model to address the problem at the individual level, especially in settings where specialized hepatology services are not in high demand (34, 37, 38). These methods can be used in conjunction with new translational endeavors to link immune profiling to clinical decision-making.

In general, the clinical and translational implications of this bibliometric review support the need to integrate immunological evaluation in the treatment of long-term coinfection of HIV/HBV. The integration of antiviral treatment methods with immune-specific observations and multidisciplinary studies could contribute to achieving the following objectives: risk stratification, personalized follow-up, and, ultimately, improved long-term prognosis for individuals with HIV/HBV co-infection. These findings support a transition toward immune-informed clinical management, in which immune monitoring and immune-mediated risk stratification complement antiviral therapy in long-term HIV/HBV care.

### 4.5 Research gaps and future directions in HIV/HBV co-infection

Although the research on HIV/HBV co-infection has been growing substantially and methodologically sophisticated, several gaps highlight critical gaps in immunology research. A critical dissonance is that regions with the highest disease burden and those that generate immunological research remain disconnected. The contributions of sub-Saharan Africa and other high-burden regions to epidemiological data have been underrepresented in studies of immune mechanisms, immune phenotyping, and host-virus interactions. This imbalance indicates broader structural injustices in international health research and impedes insight into context-dependent immune responses shaped by genetic variation, co-infections, and environmental influences (39). To fill this gap, a long-term commitment to immunology-oriented research led by local authorities, together with the formation of a long-term cohort in high-burden environments, is needed to generate immune-relevant evidence that can be generalized globally (23, 34).

A second significant gap is the limited availability of long-term, immune-informed outcome data. Although newer research is starting to consider prognosis, liver morbidity, and disease burden, most of the literature published until now is based on cross-sectional designs or cohorts with mostly limited follow-up periods. There is a dearth of large-scale longitudinal studies that combine virological suppression with immune parameters, clinical outcomes, noninvasive measures of liver fibrosis, and a long duration of antiretroviral exposure. These incorporative strategies are necessary to outline the role of chronic immune activation, immune exhaustion, and chronic inflammation in the development of progressive liver disease and adverse outcomes, especially in aging populations with long-term HIV/HBV co-infection (17, 25).

Moreover, significant gaps remain in translational immunology linking immune dysfunction to clinical strategies for real-world monitoring. Although immune-mediated liver injury and inflammatory pathways are increasingly recognized as drivers of disease progression, few studies have systematically integrated immune biomarkers or immune profiling into standard longitudinal follow-up models (39). The validation of immune-informed risk stratification methods, such as those based on indicators of chronic inflammation, immune activation, and immune responses to fibrosis, should be prioritized in future research, as they can better identify individuals at high risk of liver-related complications (34, 37, 40).

Lastly, future research should focus on bridging mechanistic immunology to clinical use. The adoption of immune monitoring as a supplementary component of current HIV/HBV management paradigms could allow for detecting the immune-driven disease progression earlier and create an adjunctive treatment approach to immune activation along with antiviral therapy (41). The future of immunology-based, longitudinal, and translational research will be significant to enhance the outcome and align future research with the changing clinical realities of HIV/HBV co-infection.

### 4.6 Strengths and limitations

There are several strengths of this study. First, it combines data from two large bibliographic databases, thereby providing a general overview of research on HIV/HBV coinfections worldwide. Second, it is evident that structural collaboration networks, thematic patterns, and time-specific research trends can be systematically visualized using a variety of bibliometric methods, thereby providing a reproducible mapping of the knowledge landscape.

Several limitations should also be considered. It included only English-language publications, which may have resulted in underrepresentation of relevant research from non-English-speaking regions, including several high-burden settings. Additionally, citation-based indicators may favor older publications that have had more time to accrue citations, potentially understating the impact of more recent, high-quality studies.

Despite the use of two large databases, not all regional journals and other research outputs may have been indexed, potentially leading to a geographic imbalance in publication distribution. Lastly, because this study is a bibliometric analysis, it cannot assess the methodological quality of individual studies or causal relationships. Instead, it provides a descriptive review of research trends and findings that must be viewed in relation to systematic reviews and primary studies that address clinical and implementation questions.

## 5 Conclusions

This bibliometric review provides an in-depth overview of the current state of knowledge on HIV/HBV coinfection over the past decade. The results indicate a continued increase in research output and a clear shift in thematic focus toward long-term outcomes, liver disease progression, and prognosis. These patterns are indicative of the evolving clinical reality of comorbidity between HIV/HBV in the age of effective antiretroviral therapy.

The discussion also highlights the central role of a few countries and institutions in shaping research agendas, as well as the growing involvement of high-burden regions in international collaboration. Thematic evolution also signals an increased focus on long-term management, aging populations, and risk stratification, as well as the necessity of addressing immune-mediated liver complications beyond required viral suppression.

Overall, this paper traces the evolution of research on HIV/HBV coinfection and outlines new priorities, providing a reference for subsequent studies. The results are in favor of more balanced international research leadership, longitudinal research, and implementation-driven research to enhance clinical care and health outcomes in people with HIV/HBV co-infection.

Future research integrating immunological mechanisms with longitudinal clinical outcomes will be essential to advance immune-informed prevention and management strategies for HIV/HBV co-infection.

## Data Availability

The original contributions presented in the study are included in the article/supplementary material, further inquiries can be directed to the corresponding author.
